# The U.S. diabetes belt and factors explaining the excess risk: Multifactorial modeling and machine learning analysis^☆^

**DOI:** 10.1016/j.pcd.2025.12.001

**Published:** 2025-12-11

**Authors:** Longjian Liu, Nathalie S. May, Yuwei Hou, Jingyi Shi, Edward J. Gracely, Arthur L. Frank, Howard J. Eisen

**Affiliations:** a Department of Epidemiology and Biostatistics, Dornsife School of Public Health, Drexel University, Philadelphia, PA 19104, United States; b Department of Medicine, College of Medicine, Drexel University, Philadelphia, PA 19104, United States; c Center for Center for Biomedical Informatics and Genomics, John W. Deming Department of Medicine, School of Medicine, Tulane University, New Orleans, LA 70112, United States; d Department of Mathematics and Statistics, Mississippi State University, Starkville, MS, USA; e Department of Family, Community & Preventive Medicine, College of Medicine, Drexel University, Philadelphia, PA 19104, United States; f Department of Environmental and Occupational Health, Dornsife School of Public Health at Drexel University, Philadelphia, PA 19104, United States; g Clinical Research for the Advanced Cardiac and Pulmonary Vascular Disease Program, Thomas Jefferson University Hospital, Philadelphia, PA 19107, United States

**Keywords:** Geographic variation, Diabetes mellitus, Risk factors

## Abstract

**Aim:**

Research on the epidemiology of diabetes mellitus (DM) has identified a geographically distinct region in the United States (U.S.) known as the diabetes belt (DM Belt), which represents a significant public health concern. This study aimed to examine the factors contributing to the increased risk of DM in the DM Belt compared to the non-DM Belt.

**Methods:**

Data were analyzed from 398,243 adults aged ≥ 18 years who participated in the 2019 Behavior Risk Factor Surveillance System. DM status was based on participants’ self-reported physician-diagnosed DM. The DM Belt was defined at the state level according to the U.S. Center for Disease Control and Prevention’s classification. Logistic regression (LR) was used to estimate odds ratios for DM and assess the excess DM risk in the DM Belt versus the non-DM Belt. Random Forest (RF) and stepwise LR were employed to identify and rank key contributors to the excess DM risk.

**Results:**

Residents of the DM Belt had a significantly higher prevalence of DM than those in the non-DM Belt (age-sex-adjusted rate: 12.5 % versus 10.5 %, p < 0.001). Low socioeconomic status (SES), physical inactivity, and hypertension were identified as the top three factors explaining the excess DM risk in the DM Belt.

**Conclusions:**

These findings underscore the importance of an integrated approach to improving SES, promoting healthy behaviors, managing chronic conditions for reducing DM risk. Addressing these factors can help mitigate health disparities in DM risk across the U.S.

## 1. Introduction

Diabetes mellitus (DM) ranks as the eighth leading cause of death in the United States (U.S.). In 2019, approximately 37.3 million people, accounting for 11.3 % of the US population, had DM, including both diagnosed and undiagnosed cases. Of these individuals, 37.1 million were adults aged 18 or older, comprising 14.7 % of all U.S adults. About 8.5 million of these adults had DM but were either unaware of their condition or did not report it [1]. Over the years, there has been a notable decline in the incidence of diagnosed cases, with rates dropping from an age-adjusted rate of 8.4 per 1000 adults in 2008 to 5.9 per 1000 adults in 2019 [2]. However, the prevalence of DM, which encompasses all existing cases (both prevalent and incident cases), has shown a significant increase in the U.S. This rise can be attributed in part to improved self-management practices and health care services, leading to individuals living longer with DM. Notably, the U.S. has observed a geographic concentration of DM known as the diabetes belt (DM Belt), predominantly situated in the Southern states. These states bear a considerably higher burden of DM-related health issues [3,4]. However, the determinants of this geographic disparity have yet to be studied in detail. The present study aimed to investigate DM risk factors that may explain this excess burden of DM in the DM Belt compared to the non-DM Belt. We hypothesized that socioeconomic disadvantages, unhealthy lifestyle factors, and chronic conditions would significantly explain this elevated DM risk in the DM Belt.

## 2. Methods

### 2.1. Data and study population

We analyzed data from the 2019 Behavior Risk Factor Surveillance System (BRFSS), the nation’s premier system of health-related telephone surveys that collect state-level data about U.S. residents regarding their health-related risk behaviors, chronic health conditions, and use of preventive services [5]. Among the total participants aged ≥ 18 years (n = 418,268) in the 2019 BRFSS, we excluded 2504 women who had gestational diabetes (n = 2122) and those who reported uncertain whether they had gestational diabetes (n = 201), and those who refused to answer whether they had gestational diabetes (n = 181). We further excluded participants with missing values on the classification of their DM status (N = 9485), and those with missing values on their residential status (n = 8036). The final study sample size was 398,243, which represents 95.2 % of the total participants in the 2019 BRFSS.

Data from the BRFSS is de-identified and publicly available for research. We received a review approval from Drexel University Institutional Review Board (IRB# 2302009773) for conducting this study.

### 2.2. Measures

#### Exposures:

(1) Definition of DM Belt: In 2010, the U.S. Center for Disease Control and Prevention (CDC) scientists identified a diabetes belt in 644 counties in 15 states, located mostly in the southern portion of the U.S. These 15 states are Alabama, Arkansas, Florida, Georgia, Kentucky, Louisiana, Mississippi, North Carolina, Ohio, Pennsylvania, South Carolina, Tennessee, Texas, Virginia, and West Virginia [6]. We defined the DM Belt by including these 15 states instead of county levels because BRFSS data have state record only. The remaining states (n = 36, including the District of Columbia) are classified as non-DM Belt. (2) Factors that may explain the variations in diabetes risk between DM Belt and no-DM Belt: (a) demographic and SES: age (years), gender (males and females), race/ethnicity (non-Hispanic [NH] White, NH Black, and all other race/ethnicity groups), and residential status (urban or rural based on the metropolitan statistical areas in the BRFSS). SES was assessed from three indictors: health insurance coverage, educational attainments (<high school, completed high school, associate degree, and those ≥ college degree), and household average annual income. (b). Lifestyle-related factors: body mass index (BMI), calculated by weight (kg)/height squared (m^2^), cigarette smoking (current smokers, former smokers, or never-smoked), physical activity, and vegetable and fruit intake (number of servings per day). Physical activity was categorized into four groups: (i) inactive (no physical activity during the past month); (ii) insufficiently active (1–149 min of physical activity per week); (iii) Active (150–300 min or vigorous equivalent of physical activity per week); and (vi) highly active (>300 min (or vigorous equivalent) aerobic recommendation) [7]. (c) Chronic conditions included hypercholesterolemia and hypertension, defined by participants’ self-report of physician-diagnosed conditions [8,9].

#### Outcome:

Diabetes cases were classified based on participants’ self-report of physician-diagnosed diabetes and those who were under anti-diabetic treatment.

### 2.3. Statistical analysis

We performed four sets of data analyses. First, we performed univariable analysis of the characteristics of participants by the DM Belt status. The difference in continuous variables was tested using student’s *t*-test and in categorical variables was tested using Chi-square test. Second, we estimated age- and sex-adjusted prevalence of DM using standard adjusted rate method, and odds ratios (ORs) for DM associated with SES, lifestyle, and chronic conditions factors using logistic regression analysis. Third, to estimate the excess risk of DM in the DM Belt versus the non-DM Belt, we tested three groups of factors: (1) SES, (2) lifestyle factors, and (3) chronic conditions. In the logistic regression analysis, we applied a step-by-step approach to include and test covariates. Model 1 is the base model to estimate age- and sex-adjusted OR (95 %CI) for DM in the DM Belt versus the non-DM Belt. In model 2, we included race as a covariate to test whether and how much change of the estimated OR (95 %) for DM because of the inclusion (i.e., the change in the value of OR indicating the impact of race on the odds of DM). Model 3 adjusted residential status (urban versus rural). Model 4 included SES factors. Model 5 included lifestyle factors. Finally, in Model 6, we included chronic conditions (hypertension and hypercholesterolemia). To determine the excess risk of DM associated with these adjusted covariates we estimated the percent change of the ORs of DM using the formula: percent of excess risk of DM in the DM Belt versus the non-DM Belt attributable to these explanatory covariates = (OR_1_ - OR_2_) / (OR_1_ −1.0) × 100 %, where OR_1_ represents the OR derived from the basic model (model 1). OR_2_ to OR_6_ were the ORs after adjusting for these additional covariates [3,10]. Fourth, we applied Machine Learning (ML) algorithm (Random Forest) and logistic regression (LR) to identify important features that were associated with DM risk in DM Belt versus non-DM Belt. RF analysis, a supervised ML algorithm, constructs the result from decision tree algorithms and handles datasets with more categorical variables and potential outliers effectively, leading to better classification of the association between the study exposures and outcomes [11,12]. In the RF, a Mean Decrease Accuracy (MDA) score of individual exposures was estimated based on a 10-fold cross-validation process and a pre-set of n = 500 trees for the RF model in each fold. The imbalanced outcome cases were handled by the oversampling technique in each RF training step [13]. An MDA score indicates how much accuracy the model loses by excluding a variable. A higher MDA value signifies higher importance of the variable in the model. Using RF analysis, we ranked individual factors that explained the risk of DM in the DM Belt versus the non-DM Belt. In LR, we applied stepwise approach to rank the importance of individual factors for the purpose of comparing the results from the RF.

SAS software 9.14 (SAS Institute, Cary, North Carolina) was used in the analyses. As BRFSS applied a complex multistage and probability sampling design, we applied SAS Survey Procedures [14]. to estimate the sampling weighted means, rates, odds ratios (OR), and OR 95 %CI. Random forest analysis was performed using RStudio (version 2023.06.0 +421) with R version 4.3.0 (CRAN package Random Forest, version 4.7–1.1).

## 3. Results

### 3.1. Characteristics of the participants

The study included 398,243 participants, with 117,240 (representing 105 million adults) living in the DM Belt and 281,003 (representing 136 million adults) living in the non-DM Belt. Individuals in the DM Belt had a significantly higher DM prevalence (12.8 %, 95 %CI:12.4 %−13.1 %) than those in the non-DM Belt (10.9 %, 95 %CI: 10.7 %−11.2 %). Table 1 shows that compared to the non-DM Belt, participants in the DM Belt were, on average, older and had a greater proportion of NH Black individuals. They were also characterized by lower SES and less healthy behaviors, less likely to live in urban areas and have health insurance, have post-high school education, and more likely to have an income below $75,000, higher obesity rate and current smoking. Additionally, DM Belt residents reported less frequent physical activity and vegetable/fruit consumption and exhibiting higher rates of hypercholesterolemia and hypertension.

### 3.2. Risk of DM and interaction associated with DM belt status

The analyses in Supplementary Table 1 revealed variations in the age- and sex-adjusted odds ratios (ORs) for DM associated with socio-demographic, lifestyle, and chronic conditions across both the non-DM Belt and the DM Belt. Importantly, the magnitudes of these associations were substantially attenuated when fully adjusted for all covariates. The fully adjusted models in Table 2 highlighted differences and similarities in the factors driving DM risk in the two regions. In the non-DM Belt, factors significantly associated with an increased DM risk were race, health insurance status, high BMI, smoking, hypercholesterolemia, and hypertension. Conversely, higher education levels, household income, daily physical activity, and vegetable/fruit intake were associated with a reduced DM risk (test for trend, p < 0.05 or p < 0.001). The DM Belt exhibited findings similar to the non-DM Belt, with one key exception: the protective association between vegetable/fruit intake and DM risk was not statistically significant (test trend, p = 0.30).

Crucially, the strengths of the associations differed between the two regions. Tests for interaction confirm that the associations for race, urbanicity, and vegetable/fruit intake with DM risk were significantly stronger in the Non-DM Belt (p < 0.05).

### 3.3. Excess risk of DM in the DM belt versus the non-DM belt

Table 3 evaluates the excess risk of DM in the DM Belt versus the non-DM Belt by quantifying the contribution of various factors through sequential adjustment process. Model 1 (the base model), adjusted for age and sex, showed that DM Belt residents had a 22 % higher risk of DM (OR: 1.221, 95 %CI: 1.17–1.27). We then quantified the contribution of various factors to this excess risk by assessing the changes in the ORs. Model 2, adjusting for race, reduced the OR to 1.209, suggesting that the different distributions of the proportions by race between the two Belts accounted for 5.4 % of the excess DM risk. Model 3 (urbanity), further adjusting for residential status reduced the OR to 1.204, bringing the cumulative explained excess risk to 7.7 %. Model 4, adjusting for SES factors, significantly decreased the OR to 1.174. SES alone explained 13.6 % of the excess risk reduction, resulting in a total cumulative explained risk of 21.3 %. Model 5, adjusting for lifestyle factors, substantially reduced the OR, with lifestyle independently accounting for 16.7 % of the reduction and raising the cumulative explained risk to 38.0 %. Model 6, the final model, which included controlling chronic conditions, accounted for 55.7 % of the total excess DM risk, and explained an additional 17.7 % of the excess risk.

### 3.4. Ranking of the importance of individual factors

**Fig. 1.**
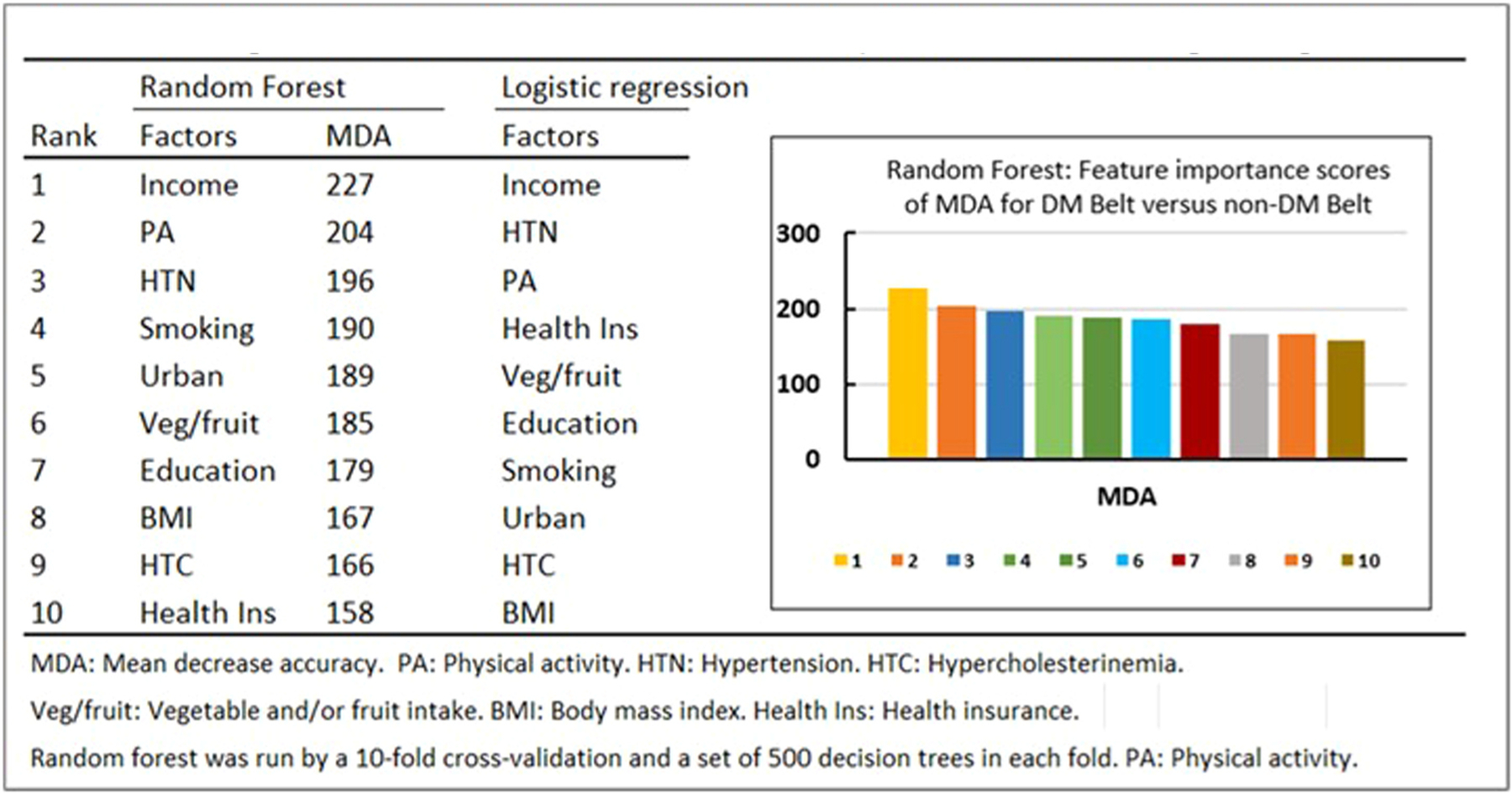


While Table 3 evaluated the excess risk of DM by three grouped categories (SES, lifestyle and chronic conditions), we were also interested in ranking the individual factors that explain the differences in DM risk between the DM Belt and non-DM Belt. This ranking was conducted using Random Forest (RF) and stepwise logistic regression. As illustrated in Fig. 1, both modeling approaches consistently identified the same three factors as the most important predictors of the excess DM risk. This included household income, physical activity, hypertension in RF, and household income, hypertension, and physical activity in LR. Although the top three factors were largely the same, the ranking of the remaining predictors showed clear divergence between the two modeling approaches.

## 4. Discussion

Our findings confirm a substantial disparity in diabetes prevalence across the United States. This study showed that the DM Belt has a significantly higher prevalence of DM (12.8 %, 95 %CI: 12.4–13.1) compared to the non-DM Belt (10.9 %,95 %CI:10.7–11.2). When adjusting for age and sex, participants residing in the DM Belt faced a 22 % higher risk of DM (OR: 1.22, 95 %CI: 1.17–1.27). Our subsequent sequential adjustment analysis indicated that an estimated 55.7 % of this excess DM risk between the DM Belt and non-DM Belt could be attributed to the geographic differences in the distribution of race, urbanicity, SES, lifestyle factors, and chronic conditions.

Diabetes unequally affects the American population, with a much higher prevalence among ethnic minority groups and those living in the southern region of the U.S. In 2011, the U.S. CDC scientists identified this as a diabetes belt, a region of counties (n = 644) in 15 states, mostly located in the Southern U.S [3,4,6]. Several studies have investigated factors attributable to DM risk [3,15,16]. Myers and colleagues conducted a county-level ecological study and observed that in counties in the non-DM Belt there were stronger positive associations between diabetes and economic hardship (poverty and unemployed status). However, in counties within the DM Belt, diabetes prevalence showed a stronger negative association with fitness and recreation facility density. Lobo and colleagues examined county-level data (n = 3129) from the Small Area Health Insurance Estimates and Area Health Resources Files (2012–2016) and observed that the Patient Protection and Affordable Care Act (which expanded Medicaid in the U.S. in March 2010, and most were phased in and took full effect in January 2014, known as “Obamacare”) was strongly associated with a reduced uninsured rate, leading to a decreased DM rate in the DM Belt compared to those in the non-DM Belt [16]. These previous studies addressed the burden of DM and its correlation with mean county-level SES instead of individual level [15, 16]. Therefore, findings from the previous research were subjective to ecologic analysis bias, which refers to the limitations of using aggregated group data (i.e., mean county-level data) to represent individual measures of participants [17]. However, in the study, we examined risk factors at individual-level of 398,243 participants in the 2019 BRFSS.

Our results demonstrate that NH Black and other racial groups faced a significantly greater risk of DM than NH White individuals in both the non-DM and DM Belts. The age- and sex-adjusted prevalence rates (95 % CI) of DM were 9.6 % (9.4 %−9.8 %) in NH White and 14.7 % (13.8 %−15.7 %) in NH Black in non-DM Belt. These corresponding rates (95 % CI) were 12.0 % (11.6 %−12.3 %) in NH White and 16.1 % (15.2 %−17.0 %) in NH Black in the DM Belt. A significant interaction was observed (p = 0.035), indicating that DM Belt status modified this racial disparity. Notably, the relative risk of DM between NH Black and NH White individuals was significantly larger within the non-DM Belt than that in the DM Belt. Considering race as a social construct, this finding suggests that that the elevated risk for NH Black individuals is pervasive across the U.S. However, the greater relative difference in the non-DM Belt may imply that regional differences in social determinants of health, such as access to resources, segregation, or systemic factors, likely drive these varying racial disparities across the two regions.

Regarding other factors, the influence of urbanicity and vegetable/fruit consumption warrants closer examination. (1) Urbanicity and DM Risk: Although urbanicity was not significantly associated with DM risk in either belt in the fully adjusted model (Table 2), the significant interaction effect with DM Belt status suggests a potentially different impact of residential status across the two regions. This points to unique, underlying community-level factors within the DM Belt that may negate or modify the protective or risk effects typically associated with urban or rural settings. (2) Vegetable/Fruit Consumption and DM Risk: We also observed a significant interaction effect by DM Belt status on the association between vegetable/fruit consumption and DM risk. While the age- and sex-adjusted analysis (Supplementary Table 1) showed a clear protective effect of increased daily vegetable/fruit intake on the risk of DM, this association was significantly attenuated in the fully adjusted model, particularly within the DM Belt. This attenuation indicates that the apparent protective effect of vegetable/fruit consumption is largely explained by the co-occurrence of other covariates (such as SES and physical activity) included in the fully adjusted model. Further research is warranted to fully explore these complex, region-specific relationships and determine if targeted dietary interventions can overcome the structural challenges present in the DM Belt.

The results from the Random Forest (RF) models provided key insights into the individual factors contributing to the DM risk disparity. The RF feature importance (Mean Decrease Accuracy, MDA) identified mean household income, followed by physical inactivity, and hypertension as the top three individual factors explaining the difference in DM risk between the DM Belt and non-DM Belt. These findings imply that controlling these specific factors will be crucial for reducing the regional disparity in DM. These results extend previous research by providing a detailed quantitative ranking of the potential factors that explain the excess odds of diabetes associated with living in the DM Belt.

We applied stepwise logistic regression (LR) to validate the risk factor ranking. Overall, the top three factors selected were the same (household income, hypertension, and physical activity), lending confidence to the importance of these variables. However, the ranking of the remaining predictors showed a clear divergence. This difference is a result of the fundamental differences in how the non-linear RF model and the linear LR model quantify variable importance and account for interactions. A key advantage of using RF is its ability to capture and rank complex, non-linear effects and interactions more accurately than the linear coefficients of LR. We acknowledge that the analysis was not repeated using other Machine Learning approaches, such as XGBoost and LASSO. This was a deliberate choice aligned with our study’s primary objectives. XGBoost was excluded because its main benefit would likely be a marginal boost in predictive accuracy, whereas our primary focus was the comparative ranking of features between a linear model (LR) and a non-linear model (RF). LASSO is a regularized linear model; since we already employed LR, we anticipated LASSO would yield highly similar linear feature importance, offering limited additional clinical or methodological insight for this specific comparison. Therefore, while the exploration of diverse techniques is a valuable avenue for future work, it falls outside the scope of the present study, which was focused on understanding and ranking the structural drivers of DM risk using a robust model comparison framework.

The precise mechanisms by which SES, lifestyle factors, hypertension, and hypercholesterolemia lead to the high risk of DM and contribute to the DM Belt disparity are complex. However, several pathways are suggested: (1) SES: Individuals with better SES typically have greater receptiveness to health information and possess the resources and security to be more health conscious. This allows them to more readily adopt healthy behaviors, make effective lifestyle modifications, and utilize the healthcare system proactively [18–24]. Low SES, conversely, is associated with chronic stress, limited access to healthy foods, and poor healthcare access, all of which drive DM risk. (2) Unhealthy Lifestyle and Chronic Conditions: The unhealthy lifestyle factors identified, including increased BMI, smoking, physical inactivity, and poor vegetable/fruit intake, contribute to DM risk through established biological mechanisms. These behaviors have detrimental effects on vascular integrity, promote chronic inflammation, and directly lead to insulin resistance [8,9,23–26].

The present study has several key strengths. First, we utilized data from the 2019 BRFSS, which is one of the U.S. largest nationally population-based health surveys (n = 374,396), representing over 241 million noninstitutionalized U.S., provides exceptional statistical power and high degree of generalizability to the U.S. adult population. adults residing in the nation). Second, we applied a relatively appropriate state-level definition for DM Belt [3], which aligns with the classifications used by the CDC and facilitates a clear delineation between high-prevalence and low-prevalence regions for comparative analysis. Third, our study strategically focused on three crucial groups of preventable risk factors (SES, lifestyle, and chronic conditions), which are paramount for developing effective intervention strategies at both the population and individual levels.

Fourth, the methodology offers robust insights by integrating a standard biostatistical approach (Logistic Regression) with a Machine Learning algorithm (Random Forest, RF). The use of RF is a key strength, as it provides a superior approach to precisely identify and rank feature importance. RF achieves this by reducing potential overfitting through resampling techniques and the creation of multiple decision trees, thereby yielding a more accurate, non-linear estimation of diabetes-associated risk factors than conventional linear models. This comparative, data-driven methodology advances the understanding of this large-scale public health issue.

It is important to note that our study has several limitations, primarily related to the data source and methodology. First, The findings are based on cross-sectional analyses, a characteristic inherent to the BRFSS study design. Consequently, we are unable to determine cause- and-effect relationships between the study factors and DM risk, as temporal precedence cannot be established. Second, we defined the DM Belt status at the state level, which introduces the potential for ecological misclassification. This occurs because some states classified as part of the DM Belt include a few counties with lower DM rates than those found in the non-DM Belt. However, this state-level definition retains significant public health utility because most health policies, funding decisions, and large-scale health promotion programs in the U.S. are planned, implemented, and evaluated at the state level. The data, therefore, offer a unique and appropriate platform to evaluate and compare outcomes in the context of state-level health systems and policies. Third, the classification of DM relied on participants’ self-reported physician-diagnosed disease, which may introduce potential information bias regarding disease status. Nevertheless, this bias is likely limited, as the validity of self-reported chronic health conditions has been confirmed and supported by substantial agreement between self-reported surveys and medical records in numerous nationwide health surveys [27,28]. Fourth, the BRFSS does not include biomarker measurements, such as fasting glucose, hemoglobin A1c, and lipid profiles. Consequently, individuals with unknown chronic conditions may be classified as non-disease, which could lead to an underestimate of the prevalence of DM and the association between risk factors and DM. These limitations should be considered when interpreting the results.

## 5. Conclusions

This study, using a nationally representative population-based sample, found that the significant excess risk of diabetes in the DM Belt versus non-DM Belt regions can be explained by the integrated risk factors of lower SES, unhealthy lifestyle, and higher prevalence of hypertension and hypercholesterolemia. These comprehensive quantitative findings highlight the critical importance of improving SES, healthy behaviors, and effectively managing these conditions to reduce the risk of DM and ultimately eliminate geographic disparities in diabetes risk across the U.S.

## Supplementary Material

1

## Figures and Tables

**Table 1:** Characteristics of participants, BRFSS 2019 (N = 398,243).

	Non-DM Belt		DM Belt		
	Rate, %	(SEP)	Rate, %	(SEP)	p-value
Sample size, n	281,003		117,240		
Age, mean (SD)	55.5	(17.6)	55.9	(17.5)	< 0.001
Sex, % (SEP)					0.045
Males	50.0	(0.2)	49.3	(0.3)	
Female	50.0	(0.2)	50.7	(0.3)	
Race/ethnicity					< 0.001
NH White	63.6	(0.2)	63.0	(0.3)	
NH Black	8.2	(0.1)	16.4	(0.2)	
Others	28.2	(0.2)	20.6	(0.3)	
Urban county, yes	94.2	(0.1)	92.5	(0.1)	< 0.001
Insurance, yes	88.8	(0.1)	84.8	(0.2)	< 0.001
Education					< 0.001
< High school	4.8	(0.1)	4.5	(0.2)	
High school	33.5	(0.2)	39.0	(0.3)	
Associate degree	31.6	(0.2)	30.3	(0.3)	
≥ College	30.1	(0.2)	26.1	(0.2)	
Household income / yr.					< 0.001
< 20 K	15.4	(0.2)	17.6	(0.2)	
20 K - 34 K	17.2	(0.2)	20.3	(0.3)	
35 K - 74 K	27.7	(0.2)	28.1	(0.3)	
≥ 75 K	39.7	(0.2)	34.0	(0.3)	
BMI, Kg/m^2^					< 0.001
< 18.5	1.9	(0.1)	2.0	(0.1)	
18.5–24	33.3	(0.2)	29.6	(0.3)	
25–29	35.4	(0.2)	35.2	(0.3)	
≥ 30	29.4	(0.2)	33.2	(0.3)	
Smoking					< 0.001
Never smoked	61.4	(0.2)	58.7	(0.3)	
Formerly smoked	24.7	(0.2)	24.0	(0.2)	
Currently smoked	13.9	(0.1)	17.4	(0.2)	
Veg / fruit, Serving /day					< 0.001
0 or rare	13.8	(0.1)	14.7	(0.2)	
1–2 Serving / d	45.3	(0.2)	47.1	(0.3)	
3 Servings / d	18.8	(0.2)	18.1	(0.2)	
≥ 4–5 Servings / d	22.1	(0.2)	20.1	(0.2)	
Physical activity					< 0.001
Inactive	27.6	(0.2)	31.8	(0.3)	
Insufficiently active	20.4	(0.2)	19.8	(0.2)	
Active	18.7	(0.2)	17.4	(0.2)	
High active	33.3	(0.2)	31.1	(0.3)	
Chronic conditions					
Hypercholesterolemia	30.0	(0.2)	32.6	(0.3)	< 0.001
Hypertension	30.6	(0.2)	35.4	(0.3)	< 0.001

NH White: Non-Hispanic White. BMI: Body mass index. Veg/fruit: Vegetable or fruit intake / day.

Weighted rates (%) and mean, estimated using SAS survey procedure by taking account of the complex survey design of BRFSS. SEP: Standard error of proportion.

**Table 2:** Adjusted odds ratios (95 %CI) of potential predicators for diabetes by DM-Belt regions.

	non-DM Belt			DM Belt			Test for
	OR	(95 %CI)	p-value	OR	(95 %CI)	p-value	interaction
Race/ethnicity (Ref: NH White)							**0.035**
NH Black	1.82	(1.64–2.02)	**< 0.001**	1.54	(1.39–1.71)	**< 0.001**	
Others	1.85	(1.70–2.02)	**< 0.001**	1.47	(1.25–1.73)	**< 0.001**	
Urban							**0.034**
Yes vs. no	1.02	(0.94–1.10)	0.70	0.93	(0.84–1.03)	0.15	
Health insurance							0.78
Yes vs. no	1.46	(1.28–1.66)	**< 0.001**	1.39	(1.16–1.67)	**< 0.001**	
Education (Ref: <HS)							0.78
HS	0.80	(0.68–0.96)	**0.01**	0.79	(0.59–1.05)	0.10	
Associate	0.81	(0.68–0.96)	**0.018**	0.79	(0.59–1.07)	0.13	
≥ College	0.69	(0.57–0.82)	**< 0.001**	0.62	(0.45–0.84)	**0.002**	
Test for trend			**< 0.001**			**< 0.001**	
Household income (Ref: < [20]K)							0.70
20 K - 34 K	0.70	(0.63–0.77)	**< 0.001**	0.76	(0.67–0.86)	**< 0.001**	
35 K - 74 K	0.59	(0.54–0.65)	**< 0.001**	0.62	(0.55–0.70)	**< 0.001**	
≥ 75 K	0.48	(0.44–0.53)	**< 0.001**	0.50	(0.43–0.57)	**< 0.001**	
Test for trend			**< 0.001**			**< 0.001**	
BMI, kg/m^2^, (Ref 18.5–24)							0.39
< 18.5	0.80	(0.58–1.10)	0.16	0.59	(0.41–0.85)	**0.005**	
25–29	1.84	(1.68–2.02)	**< 0.001**	1.93	(1.68–2.21)	**< 0.001**	
≥ 30	4.58	(4.20–5.00)	**< 0.001**	4.23	(3.70–4.83)	**< 0.001**	
Test for trend			**< 0.001**			**< 0.001**	
Smoking (Ref: No)							0.33
Formerly smoked	1.18	(1.10–1.26)	**< 0.001**	1.13	(1.03–1.24)	**0.01**	
Currently smoked	1.13	(1.03–1.23)	**0.01**	1.05	(0.93–1.19)	0.41	
Test for trend			**< 0.001**			0.24	
Veg/fruit (Ref: < [1] serving/d)							**0.025**
1–2 Servings/d	0.98	(0.89–1.07)	0.66	0.98	(0.87–1.10)	0.74	
3 Servings/d	1.04	(0.93–1.15)	0.53	1.13	(0.98–1.30)	0.09	
≥ 4–5 Servings / d	0.89	(0.80–0.99)	**0.032**	1.02	(0.89–1.17)	0.79	
Test for trend			**0.023**			0.30	
Physical activity (Ref: inactive)							0.94
Insufficiently active	0.93	(0.85–1.01)	0.086	0.92	(0.82–1.05)	0.21	
Active	0.75	(0.69–0.83)	**< 0.001**	0.82	(0.71–0.95)	**0.01**	
High active	0.67	(0.62–0.72)	**< 0.001**	0.64	(0.58–0.71)	**< 0.001**	
Test for trend			**< 0.001**			**< 0.001**	
Chronic conditions							
H-TC (yes vs. no)	2.27	(2.13–2.43)	**< 0.001**	2.21	(2.02–2.42)	**< 0.001**	0.28
HTN (yes vs. no)	2.52	(2.35–2.70)	**< 0.001**	2.33	(2.11–2.58)	**< 0.001**	0.12

NH White: Non-Hispanic White. BMI: Body mass index. Veg/fruit: Vegetable or fruit intake/day. H-TC: Hypercholesterolemia. HTN: Hypertension.

OR: Odds ratios were estimated using SAS Proc Survey logistic regression procedures.

Full adjusted age, sex, and factors in the Table, adjusted each other, except for H-TC and HTN because they were potential mediators.

Test interaction effect of individual factors and DM Belt (yes vs. non) on DM Risk. Interaction term = factor*DM Belt.

**Table 3:** Odds ratios (OR) and 95 %CI for the likelihood of diabetes.

In DM Belt versus non-DM Belt				
				% of excess
		DM Belt vs. non-DM Belt		DM risk
Models	Controlled covariates	OR	(95 %CI)	accounted for
M1	Base model: Age, sex	1.221	(1.17–1.27)	
M2	M1 + Race/ethnicity	1.209	(1.16–1.26)	5.43
M3	M2 + Urban	1.204	(1.15–1.26)	7.69
M4	M3 + SES	1.174	(1.12–1.23)	21.27
M5	M4 + lifestyle related	1.137	(1.08–1.20)	38.01
M6	M5 + HTC and HTN	1.098	(1.04–1.16)	55.66

SES: Socioeconomic status, measured by Insurance, education, annual house-hold income. Lifestyle: BMI, smoking, vegetables and fruit intake, and physical activity. HTC: Hypercholesterinemia. HTN: Hypertension % of the excess diabetes risk in DM Belt vs. non-DM Belt, was accounted for by controlling covariates using SAS survey logistic regression model.

## Appendix A. Supporting information

Supplementary data associated with this article can be found in the online version at doi:10.1016/j.pcd.2025.12.001.
