# Supplementary material for: The U.S. diabetes belt and factors explaining the excess risk: Multifactorial modeling and machine learning analysis
Source: Prim Care Diabetes. Author manuscript; Available in PMC 2026 Jul 28. (PMC13410521; doi:10.1016/j.pcd.2025.12.001)
Supplement: 1 [file NIHMS2187930-supplement-1.docx]

| **Supplementary Table** | |  |  |  |  |  |  |  |  |  |  |  |
| --- | --- | --- | --- | --- | --- | --- | --- | --- | --- | --- | --- | --- |
| S-Table 1**.** Age-sex-adjusted OR (95%CI) of potential predicators for diabetes by DM Belt regions | | | | | | | | | | | | |
|  |  | **non-DM Belt** | | | | |  | **DM Belt** | | | | |
|  |  | OR | (95%CI) | | | p-vale |  | OR | (95%CI) | | | p-vale |
| Race/ethnicity (Ref: NH White) | |  |  |  |  |  |  |  |  |  |  |  |
|  | NH Black | 2.22 | (2.04 | - | 2.42) | <0.001 |  | 1.89 | (1.75 | - | 2.05) | <0.001 |
|  | Others | 2.13 | (2.00 | - | 2.28) | <0.001 |  | 1.60 | (1.42 | - | 1.80) | <0.001 |
| Urban | |  |  |  |  |  |  |  |  |  |  |  |
|  | Yes vs. no | 1.02 | (0.95 | - | 1.08) | 0.66 |  | 0.82 | (0.76 | - | 0.88) | <0.001 |
| Health insurance | |  |  |  |  |  |  |  |  |  |  |  |
|  | Yes vs. no | 0.92 | (0.83 | - | 1.03) | 0.13 |  | 1.09 | (0.95 | - | 1.25) | 0.21 |
| Education (Ref: <HS) | |  |  |  |  |  |  |  |  |  |  |  |
|  | HS | 0.53 | (0.47 | - | 0.59) | <0.001 |  | 0.65 | (0.54 | - | 0.78) | <0.001 |
|  | Associate | 0.45 | (0.40 | - | 0.50) | <0.001 |  | 0.56 | (0.46 | - | 0.67) | <0.001 |
|  | >=College | 0.28 | (0.25 | - | 0.32) | <0.001 |  | 0.35 | (0.29 | - | 0.42) | <0.001 |
| Household income (Ref: <20K) | |  |  |  |  |  |  |  |  |  |  |  |
|  | 20K - 34K | 0.66 | (0.61 | - | 0.72) | <0.001 |  | 0.71 | (0.64 | - | 0.80) | <0.001 |
|  | 35K - 74K | 0.50 | (0.47 | - | 0.54) | <0.001 |  | 0.55 | (0.50 | - | 0.61) | <0.001 |
|  | ≥75K | 0.35 | (0.32 | - | 0.37) | <0.001 |  | 0.39 | (0.35 | - | 0.43) | <0.001 |
| BMI, kg/m^2^, (Ref 18.5-24) | |  |  |  |  |  |  |  |  |  |  |  |
|  | <18.5 | 0.85 | (0.65 | - | 1.11) | 0.23 |  | 0.77 | (0.57 | - | 1.05) | 0.10 |
|  | 25-29 | 1.83 | (1.69 | - | 1.99) | <0.001 |  | 1.94 | (1.74 | - | 2.16) | <0.001 |
|  | ≥30 | 4.64 | (4.29 | - | 5.01) | <0.001 |  | 4.68 | (4.22 | - | 5.19) | <0.001 |
| Smoking (Ref: No) | |  |  |  |  |  |  |  |  |  |  |  |
|  | Formerly smoked | 1.20 | (1.14 | - | 1.26) | <0.001 |  | 1.15 | (1.07 | - | 1.24) | <0.001 |
|  | Currently smoked | 1.25 | (1.16 | - | 1.35) | <0.001 |  | 1.12 | (1.02 | - | 1.23) | 0.024 |
| Veg/fruit (Ref: <1 serving/d) | |  |  |  |  |  |  |  |  |  |  |  |
|  | 1-2 Servings/ d | 0.78 | (0.73 | - | 0.84) | <0.001 |  | 0.83 | (0.76 | - | 0.91) | <0.001 |
|  | 3 Servings/ d | 0.73 | (0.67 | - | 0.80) | <0.001 |  | 0.82 | (0.73 | - | 0.92) | 0.001 |
|  | ≥ 4-5 Servings / d | 0.58 | (0.53 | - | 0.63) | <0.001 |  | 0.69 | (0.62 | - | 0.77) | <0.001 |
| Physical activity (Ref: inactive) | |  |  |  |  |  |  |  |  |  |  |  |
|  | Insufficiently active | 0.76 | (0.72 | - | 0.81) | <0.001 |  | 0.79 | (0.72 | - | 0.87) | <0.001 |
|  | Active | 0.61 | (0.57 | - | 0.65) | <0.001 |  | 0.64 | (0.57 | - | 0.72) | <0.001 |
|  | High active | 0.47 | (0.45 | - | 0.50) | <0.001 |  | 0.47 | (0.43 | - | 0.51) | <0.001 |
| Chronic conditions | |  |  |  |  |  |  |  |  |  |  |  |
|  | H - TC (yes vs. no) | 2.92 | (2.77 | - | 3.07) | <0.001 |  | 2.91 | (2.72 | - | 3.13) | <0.001 |
|  | Hypertension (yes vs. no) | 4.02 | (3.80 | - | 4.26) | <0.001 |  | 3.95 | (3.65 | - | 4.28) | <0.001 |
| NH White: Non-Hispanic White. BMI: Body mass index. Veg/fruit: Vegetable or fruit intake / day. H-TC: Hypercholesterolemia. | | | | | | | | | | | | |
| OR: Odds ratios were estimated using survey logistic regression models by taking account the complex survey design of BRFSS. | | | | | | | | | | | | |
| Adjusted for age and sex. | |  |  |  |  |  |  |  |  |  |  |  |
